# Non‐vitamin K oral anticoagulants versus vitamin K antagonists in post transcatheter aortic valve replacement patients with clinical indication for oral anticoagulation: A meta‐analysis

**DOI:** 10.1002/clc.23793

**Published:** 2022-02-22

**Authors:** Yi‐feng Chen, Fei Liu, Xi‐wen Li, Hou‐jing Zhang, Yi‐ge Liu, Lu Lin

**Affiliations:** ^1^ Department of Pharmacy, Xiamen Cardiovascular Hospital of Xiamen University School of Medicine, Xiamen University Xiamen China; ^2^ Department of Cardiovascular Surgery, Xiamen Cardiovascular Hospital of Xiamen University School of Medicine, Xiamen University Xiamen China

**Keywords:** atrial fibrillation, meta‐analysis, oral anticoagulants, transcatheter aortic valve replacement

## Abstract

**Background:**

Current guidelines recommend oral anticoagulation (OAC) following transcatheter aortic valve replacement (TAVR) in patients with clinical indication, but the optimal antithrombotic regimen remains uncertain. We aimed to compare the efficacy and safety of non‐vitamin K oral anticoagulants (NOACs) versus vitamin K antagonists (VKAs) in patients undergoing TAVR with concomitant indication of OAC.

**Hypothesis:**

Comparing with VKAs therapy, NOACs are similar in reducing the all‐cause mortality and major bleeding in post‐TAVR patients requiring OAC medication.

**Methods:**

We searched the databases of PubMed, Embase, and Cochrane library databases to identify studies that investigated NOACs versus VKAs after TAVR in patients with another indication of OAC, which were published before 28th September 28, 2021. The effectiveness of outcomes was all‐cause mortality and stroke or systemic embolism, while the main safety outcome was major and/or life‐threatening bleeding. The hazard ratio (HR) with 95% confidence interval (CI) was used as a measure of treatment effect.

**Results:**

Our search identified eight studies. We included 4947 post‐TAVR patients with another indication of OAC allocated to the NOAC (*n* = 2146) or VKA groups (*n* = 2801). There were no significant differences in the all‐cause mortality (HR: 0.91, 95% CI: 0.77–1.08, *p* = .29, *I* ^2^ = 47%), stroke or systemic embolism (HR: 0.96, 95% CI: 0.68–1.37, *p* = .84, *I* ^2^ = 0%), and major and/or life‐threatening bleeding (HR: 1.09, 95% CI: 0.89–1.32, *p* = .40, *I* ^2^ = 30%) in both groups.

**Conclusion:**

Among post‐TAVR patients who required OAC therapy, NOACs therapy compared to VKAs is similar in reducing the all‐cause mortality, stroke or systemic embolism, and major and/or life‐threatening bleeding events.

## 1 INTRODUCTION

With recent improvements in technology, transcatheter aortic valve replacement/implantation (TAVR/TAVI) has developed into an available treatment for not only high surgical risk patients with severe symptomatic aortic stenosis (AS) but also intermediate and low surgical risk patients with AS.^1^, ^2^, ^3^, ^4^ During clinical practice, more than 30% of patients who underwent TAVR are needed to maintain long‐term oral anticoagulation (OAC) treatment, mostly due to atrial fibrillation (AF), and around 9% of patients with early post‐TAVR will develop an additional AF, which leads to the increasing number of patients after TAVR with OAC.^5^, ^6^, ^7^

Many national society and expert groups recommend oral anticoagulants for patients post‐TAVR with an indication for permanent OAC,^8^, ^9^, ^10^, ^11^ but specific anticoagulant regimens in the post‐TAVR setting still remain unclear. The choice of non‐vitamin K oral anticoagulants (NOACs) or vitamin K antagonists (VKAs) is usually debated and based on the local expert opinion because of the lack of evidence to guide the post‐TAVR anticoagulation. The previous study had shown that the NOAC group was significantly related to a lower rate of all‐cause mortality compared with the VKA group (hazard ratio [HR]: 0.53, 95% confidence interval [CI]: 0.29–0.96, *p* = .04) in patients with AF after a successful TAVR. The rates of life‐threatening or major bleeding were comparable between the two groups.^12^ However, a multicenter, open‐label, randomized controlled trial (RCT) indicated that edoxaban in AF patients who underwent TAVR was noninferior to VKAs for a composite outcome of adverse clinical events (HR: 1.05, 95% CI: 0.85–1.31, *p* = .01 for noninferiority), and the incidence of major bleeding was higher with the edoxaban than with the VKAs (HR: 1.40, 95% CI: 1.03–1.91, *p* = .93 for noninferiority).^13^ Therefore, we performed a meta‐analysis of studies for comparing the efficacy and safety of NOACs versus VKAs in post TAVR patients, requiring OAC therapy.

## 2 METHODS

### 2.1 Search strategy

The PubMed, Embase, and Cochrane Library databases were systematically searched for pertinent studies published before September 28, 2021 using the following words: “DOAC OR NOAC OR anticoagulants OR edoxaban OR apixaban OR rivaroxaban OR dabigatran” AND “bioprosthesis OR transcatheter aortic valve OR TAVI OR TAVR.” This review was conducted based on the preferred reporting items for systematic reviews and meta‐analyses (PRISMA) guidelines. We retrieved data according to the PICOS framework: Population, patients with TAVR/TAVI; Intervention, NOAC (i.e., apixaban, edoxaban, dabigatran, and rivaroxaban); Comparison, VKA or warfarin; Outcome, all‐cause mortality or death, stroke, systemic embolism, major or life‐threatening bleeding; and Study type, RCTs or observational studies. There were no restrictions on publication year or language. Additionally, we searched for recent major cardiovascular meetings and ClinicalTrials.gov for further potential information.

### 2.2 Data extractions and risk of bias assessment

Two independent reviewers (L. L. and Y. F. C.) extracted relevant data. The following data were recorded from each trial: experimental design, basic information (sample size, mean age, proportion of males, proportion of AF, CHA_2_DS_2_‐VASc score, HAS‐BLED score, STS score, duration of follow‐up, combination with antiplatelet therapy, and definition of outcomes), and original data (HRs of outcomes). The quality assessment of the studies was done independently by the two authors using the Cochrane Collaboration risk of bias 2.0 tool^14^ for RCTs and the Newcastle–Ottawa Scale for observational studies.^15^ Any disagreement was resolved by consensus among all the authors.

### 2.3 Summary measures

The primary outcomes were all‐cause mortality and stroke or systemic embolism. The safety of outcome was major and/or life‐threatening bleeding. Stroke or systemic embolism was defined as transient ischemic attack (TIA), ischemic stroke, systemic embolism, arterial thromboembolism, and cerebrovascular events. We accepted the definition of major and/or life‐threatening bleeding from each study. The hazard ratios of each outcome were extracted for the meta‐analysis.

### 2.4 Statistical analysis

This meta‐analysis reported effect sizes as pooled HR and 95% CI. The *I* ^2^ statistic was used to analyze the heterogeneity. The fixed‐effect model was used when the heterogeneity was low (*I* ^2^ ≤ 25%). Otherwise, the random‐effect model was applied.^16^ The Egger's linear regression test was employed to assess for the presence or absence of publication bias.^17^ The RevMan software (version 5.4.5) and STATA version 12.0 were used for all the analysis.

## 3 RESULTS

### 3.1 Study selection and study characteristics

**Figure 1:**
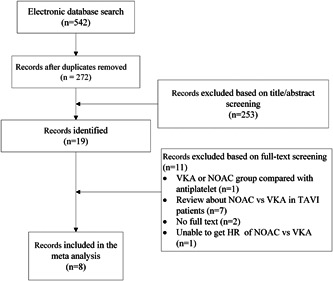
Flow chart of the included studies. NOAC, non‐vitamin K oral anticoagulant; TAVI, transcatheter aortic valve implantation; VKA, vitamin K antagonist

A total of 542 articles were found, 270 articles were excluded after duplicates removed, 259 articles were excluded after reading the titles and abstracts, three articles^18^, ^19^, ^20^ were excluded because the full texts were not published, and other two articles^21^, ^22^ were excluded because of the lack of HRs or short follow‐up duration (Figure 1). The two RCTs^13^, ^23^ and six observational studies^12^, ^24^, ^25^, ^26^, ^27^, ^28^ were initially included, and a total of 4947 patients were allocated to the NOAC (*n* = 2146) or VKA (*n* = 2801) groups.

Studies profiles and patient characteristics of eight studies were summarized in Table 1 and S1. The mean age of the participants was 80.0–84.4 years. Male patients accounted for 33.3%–58.5% of the participants. The average scores of CHA_2_DS_2_‐VASc, HAS‐BLED, and STS were 4.4–5.6, 2.7–3.3, and 4.5–8.5, respectively. The patients were high or medium risk on the STS score who benefited from the TAVR, according to the current guidelines, and had other indications for the OAC (i.e., atrial fibrillation or peripheral vascular diseases). Duration of follow‐up ranged from 1 to 3 years.

**Table 1:** Baseline characteristics of the included studies

Study or subgroup	Design	DOAC (*n*)	VKA(n)	Age (yrs)	Male (%)	AF(%)	CHA_2_DS_2_‐VASc score	HAS‐BLED score	STS score	Follow‐up
ATLANTIS 2021	RCT	223	228	N/A	N/A	N/A	N/A	N/A	N/A	1 year
ENVISAGE‐TAVI AF 2021	RCT	713	713	82.1	52.5	100.0	4.5	N/A	4.9	545 days
Butt 2019	Obs	219	516	82.0	53.7	100.0	4.9	3.3	N/A	3 years
Jochheim 2019	Obs	326	636	81.3	47.5	99.3	95.2% pts ≥2.0	N/A	4.5	593.5 days
Kalogeras 2019	Obs	115	102	82.2	58.5	64.5	N/A	N/A	N/A	15.1 months
Kawashima 2020	Obs	227	176	84.4	33.3	100.0	5.1	2.7	8.5	568 days
Mangner 2019	Obs	182	299	80.0	44.9	100.0	5.6	3.0	6.5	1 year
Seeger 2017	Obs	141	131	81.3	50.7	100.0	5.0	3.2	7.7	12 months

Abbreviations: AF, atrial fibrillation; ATLANTIS, Anti‐Thrombotic Strategy After Trans‐Aortic Valve Implantation for Aortic Stenosis; CHA_2_DS_2_‐VASc, Congestive heart failure, Hypertension, Age ≥75 years, Diabetes mellitus, prior Stroke or transient ischemic attack or thromboembolism, Vascular disease, Age 65–74 years, Sex category; DOAC, direct oral anticoagulant; ENVISAGE‐TAVI AF, Edoxaban versus Standard of Care and Their Effects on Clinical Outcomes in Patients Having Undergone Transcatheter Aortic Valve Implantation–Atrial Fibrillation; HAS‐BLED, Hypertension, Abnormal renal/liver function, Stroke, Bleeding history or predisposition, Labile international normalized ratio, Elderly (>65 years), Drugs/alcohol concomitantly; N/A, not available; Obs, observational study; pts, patients; RCT, randomized controlled trial; STS, Society of Thoracic Surgeons Predicted Risk of Mortality; VKA: Vitamin K Antagonist.

Only one study^12^ did not combine anticoagulants with antiplatelet drugs. The proportion of antiplatelet combination was not available in one study.^23^ In six other studies, the situation of concomitant antiplatelet therapy was described. In the NOAC group, apixaban was used in seven studies^12^, ^23^, ^24^, ^25^, ^26^, ^27^, ^28^; rivaroxaban and dabigatran were used in five studies^12^, ^24^, ^25^, ^26^, ^27^; and edoxaban was used in four studies.^12^, ^13^, ^26^, ^27^ The specific use of NOAC dose was found in the two RCTs^13^, ^23^ and one observational study,^28^ while other studies did not list specific medications. In the compared group, the adjusted doses of VKAs were commonly adjusted to maintain the international normalized ratio (INR) in a target range of 2.0–3.0.

Ischemic stroke, systemic thromboembolic events, and major and/or life‐threatening bleeding events were defined separately by each included study. The Valve Academic Research Consortium‐2 (VARC‐2) bleeding criteria, Bleeding Academic Research Consortium (BARC) criteria, and International Society for Thrombosis and Haemostasis definition (ISTH) were used in different studies. The included studies were of good quality and the quality evaluation is summarized in Figure S1 and Table S2.

### 3.2 Synthesis of results

**Figure 2:**
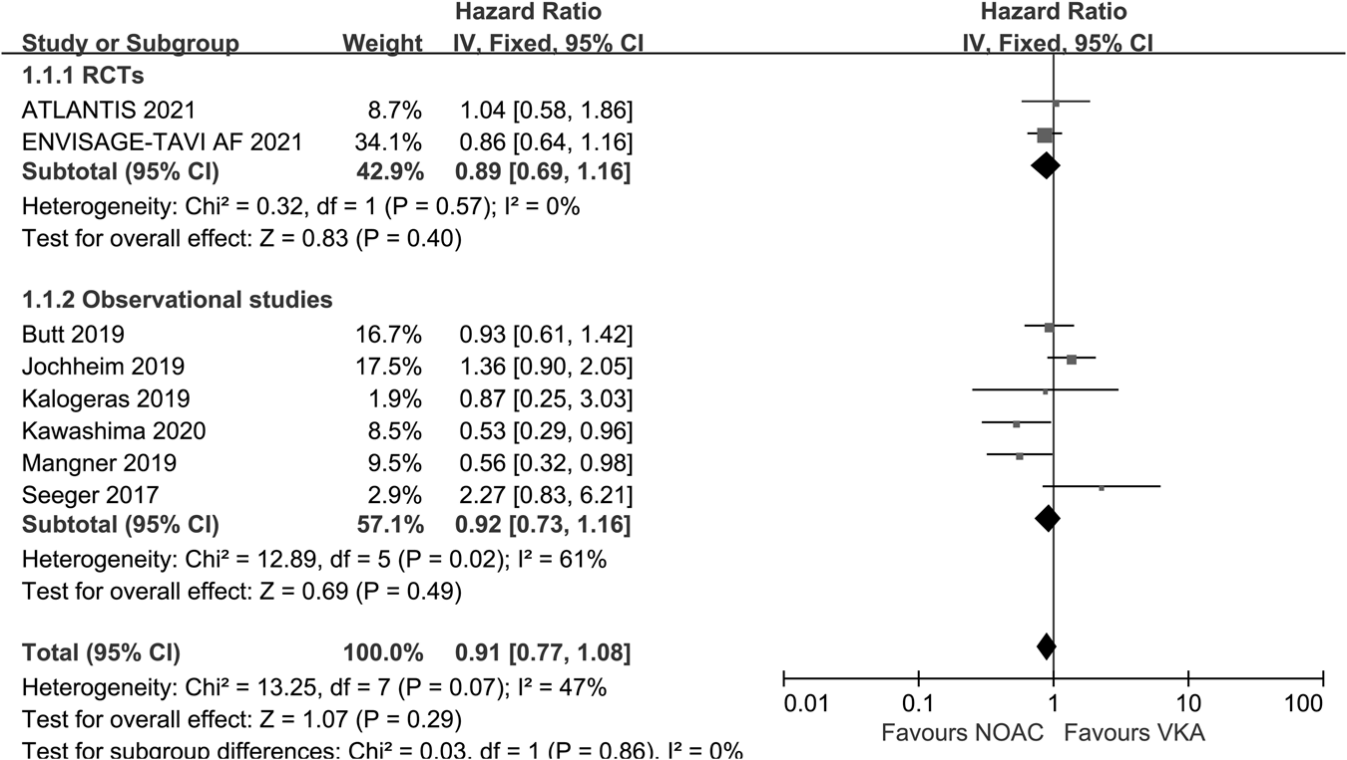
Forest plot of all‐cause mortality in post‐TAVR patients with OAC. CI, confidence; NOAC, non‐vitamin K oral anticoagulant; OAC, oral anticoagulation; RCT, randomized controlled trial; TAVR, transcatheter aortic valve replacement

**Figure 3:**
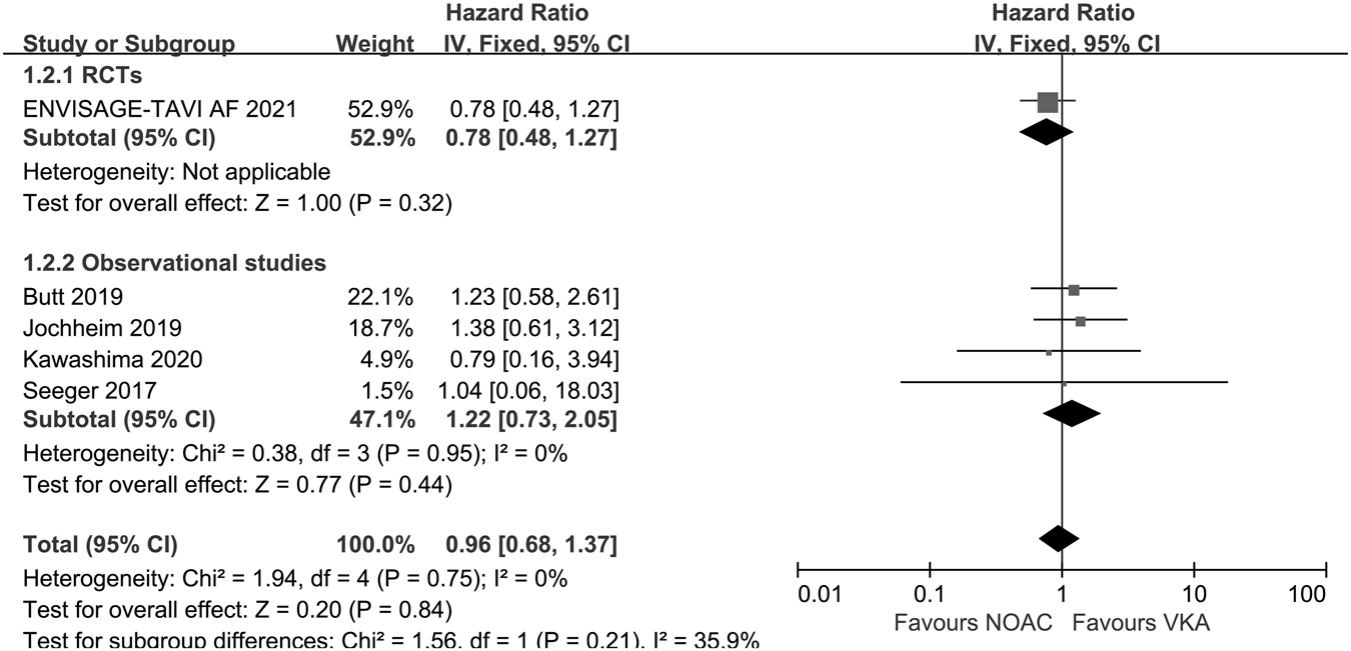
Forest plot of stroke or systemic embolism events in post‐TAVR patients with OAC. CI, confidence; NOAC, non‐vitamin K oral anticoagulant; OAC, oral anticoagulation; RCT, randomized controlled trial; TAVR, transcatheter aortic valve replacement; VKA, vitamin K antagonist

**Figure 4:**
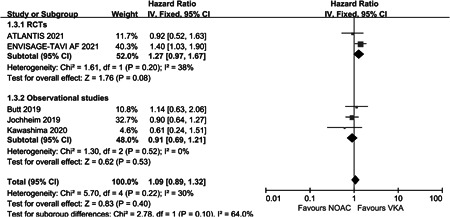
Forest plot of major and/or life‐threatening bleeding events in post‐TAVR patients with OAC. CI, confidence; NOAC, non‐vitamin K oral anticoagulant; OAC, oral anticoagulation; RCT, randomized controlled trial; TAVR, transcatheter aortic valve replacement; VKA, vitamin K antagonist

For the efficacy outcomes, there was similar between the NOAC and VKA groups for the all‐cause mortality (HR: 0.91, 95% CI: 0.77–1.08, *p* = .29, *I* ^2^ = 47%) (Figure 2). There was no significant difference in the incidence of stroke or systemic embolism events between the two groups (HR: 0.96, 95% CI: 0.68–1.37, *p* = .84, *I* ^2^ = 0%) (Figure 3). In the safety part, five studies were included in the analysis. The rate of major and/or life‐threatening bleeding was also similar between the two groups (HR: 1.09, 95% CI: 0.89–1.32, *p* = .40, *I* ^2^ = 30%) (Figure 4). All the outcomes were consistent between the included studies.

### 3.3 Bias assessment

The Egger's test did not indicate the presence of publication bias (*p* = .59, .32, and .56 for the all‐cause mortality, stroke or systemic embolism, and major and/or life‐threatening bleeding, respectively). During the sensitivity analyses, results of all the outcomes did not change by removing any single research, which showed no significant increase in the NOAC group compared to the VKA group in all the analyses.

## 4 DISCUSSION

Our findings were derived from eight studies included 4947 patients with another indication of OAC therapy. This analysis demonstrates that for post‐TAVI patients with OAC therapy, the NOACs did not increase the rates of all‐cause mortality, stroke or systemic embolism, and major and/or life‐threatening bleeding events compared with VKAs.

The findings in this study are consistent with the previous research^29^ in which five retrospective observational studies were included, enrolling 2569 participants to show similar outcomes between NOACs and VKAs in 2019. In the former study, although two of the five studies^21^, ^22^ were excluded in our analysis due to the lack of HRs for evaluating outcomes and short duration of follow‐up time, our analysis incorporated the two recent large‐scale RCTs and other three observational studies. Each study was followed‐up for more than 1 year, and the total number of participants was almost twice that of the previous analysis.

A majority of the population included in the study had AF as an indication of OAC therapy. It is well known that the AF increases the incidence of thrombosis, stroke, heart failure, and mortality, which could be reduced by appropriate antithrombotic therapy. The prevalence of pre‐existing and new‐onset AF in previous TAVR studies ranged from 15%–49% to 3%–23%, respectively.^30^ Comparing patients with pre‐existing AF and no AF, patients with new‐onset AF were related to a significantly higher risk of bleeding, stroke, and admissions from heart failure.^31^ Furthermore, a greater risk of ischemic or bleeding complications for TAVR patients with an indication of OAC could arise from the fact that most of these patients were elderly and have multiple comorbidities.

For patients with other indications for OAC in the post TAVR period, the application of OAC medication is recommended by the current guidelines and clinical practice^8^, ^9^, ^10^, ^11^; however, due to the lack of compelling evidence, there are significant inconsistencies of the OAC recommendations in this population among countries and institutions, ranging from OAC alone to triple antithrombotic therapy. It is similar to the antithrombotic regimen of each study we included, which was also different from each other. The Canadian Cardiovascular Society consensus statement recommends the NOAC therapy post‐TAVR, unless contraindicated in addition to aspirin for TAVR patients with AF. The European Society of Cardiology guidelines favor lifelong OAC for TAVR patients who have other indications for OAC, but failed to specify whether the NOAC or VKA was favored. In contrast, the updated American College of Cardiology/American Heart Association guideline mentioned that VKAs may also be considered in post‐TAVR patients with another indication for OAC after assessment of bleeding risk. Further, well‐designed randomized controlled trials regarding the antithrombotic regimen for patients post‐TAVR who require OAC therapy are warranted.

In general, NOACs have a better safety profile than VKAs, which are less likely to be influenced by food or other medications, and do not require INR monitoring. This meta‐analysis demonstrates that NOAC therapy has similar efficiency and safety compared with VKA in patients post‐TAVR, requiring OAC therapy; thus, it is an attractive alternative than VKAs, which provides important insights and evidence on OAC strategy for many patients in this setting that may inform decisions in clinical practice. Therefore, more trials are needed to conduct and study anticoagulation therapy for these specific patient populations before recommendations from guidelines can be made.

## 5 LIMITATIONS

Our study has certain limitations. First, only two studies included were RCTs and six studies were performed in a retrospective fashion, which might become a limitation to this meta‐analysis. Second, since some outcomes of subgroup data were not available, our analysis might be affected by possible selection bias. Third, most patients had AF as an indication of OAC. Patients with other indications for OAC, such as venous thromboembolism or hypercoagulability were also included, and the heterogeneity of the patient population should be taken into account when interpreting the results. Finally, different regimens in the NOAC group, various concomitant antiplatelet therapy, and follow‐up periods in each included study might limit the generalizability of the aggregate data. However, all included studies were identified as having a low risk of bias.

## 6 CONCLUSION

Our meta‐analysis suggests that in post‐TAVR patients requiring OAC therapy, all‐cause mortality, stroke or systemic embolism, and major and/or life‐threatening bleeding are similar between the NOAC and VKA groups. The NOACs may be considered as a feasible alternative to warfarin for antithrombotic treatment in this population. Future RCTs are required to further verify the conclusion.

## CONFLICT OF INTERESTS

The authors declare that there are no conflict of interests.

## Supporting information

Supporting information.

Supporting information.

Supporting information.

## Data Availability

All data, models, and code generated or used during the study appear in the submitted article.
