# Supplementary figures and images for: Non‐vitamin K oral anticoagulants versus vitamin K antagonists in post transcatheter aortic valve replacement patients with clinical indication for oral anticoagulation: A meta‐analysis
Source: Clin Cardiol. 2022 Feb 22;45(4):401–6. doi: 10.1002/clc.23793 (PMC9019885; doi:10.1002/clc.23793)

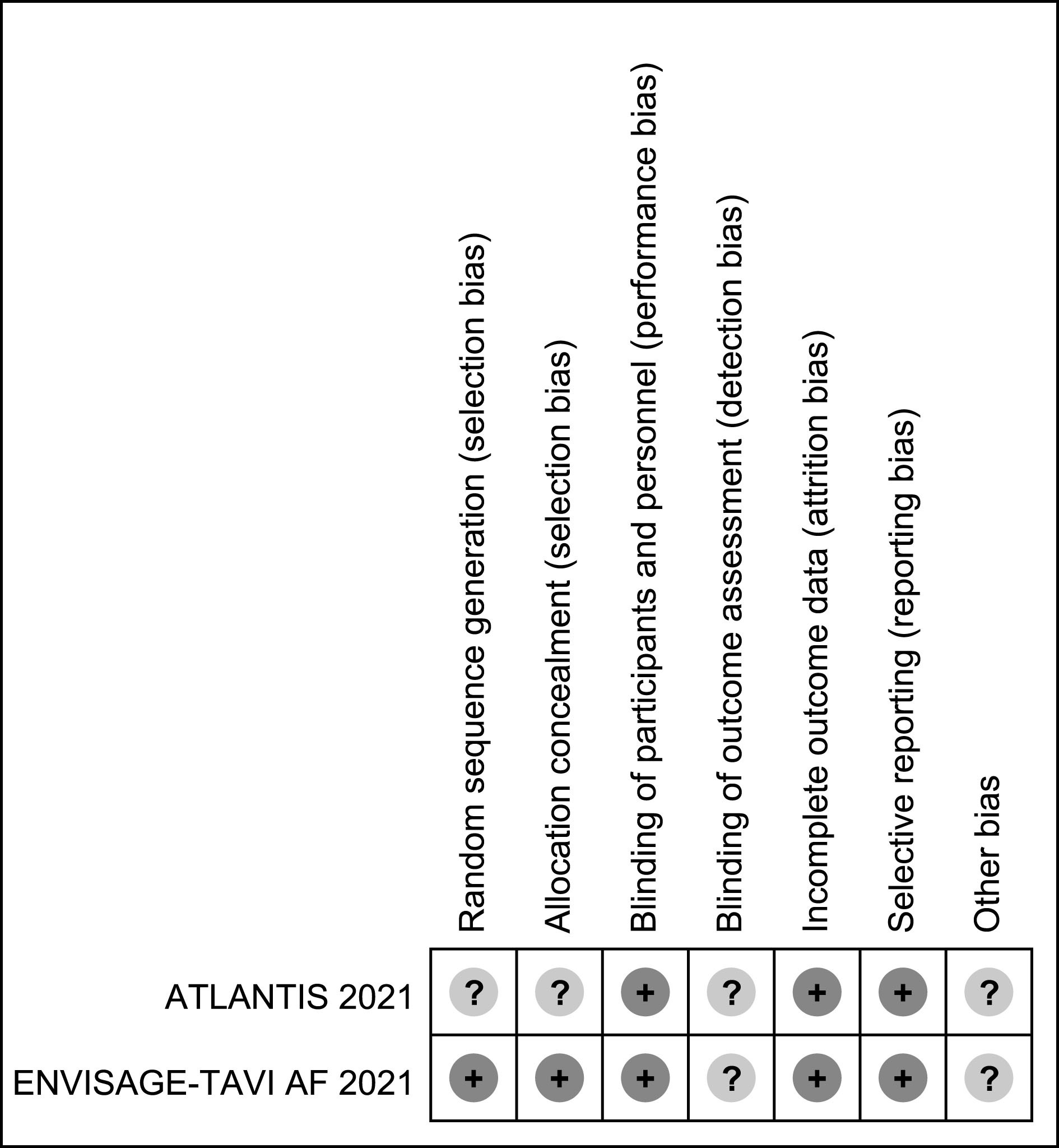

Supplement: Supplementary file 1 — Supporting information. [file CLC-45-401-s001.jpg]
