# Supplementary material for: Non‐vitamin K oral anticoagulants versus vitamin K antagonists in post transcatheter aortic valve replacement patients with clinical indication for oral anticoagulation: A meta‐analysis
Source: Clin Cardiol. 2022 Feb 22;45(4):401–6. doi: 10.1002/clc.23793 (PMC9019885; doi:10.1002/clc.23793)
Supplement: Supplementary file 2 — Supporting information. [file CLC-45-401-s002.docx]

Table S1. Summary of the antithrombotic therapy of the included studies

| **Study** | **DOAC regimen** | **Concomitant antiplatelet therapy** | **Definition of stroke or TIA or systemic embolism** | **Definition of major bleeding** |
| --- | --- | --- | --- | --- |
| **ATLANTIS 2021** | Apixaban 2.5mg/5mg twice daily | N/A | Any stroke/TIA or systemic embolism | BARC criteria |
| **ENVISAGE-TAVI AF 2021** | Edoxaban 60mg once daily | DOAC group: 46.0% received antiplatelet treatment;  VKA group: 50.4% received antiplatelet treatment. | Any stroke | ISTH definition |
| **Butt 2019** | Apixaban 38.3%; Dabigatran 30.6%; Rivaroxaban 31.1%. | DOAC group: SAPT 35.4%, DAPT 4.0%;  VKA group: SAPT 38.9%, DAPT 2.8%. | Arterial thromboembolism | Bleeding leading to a hospital admission |
| **Jochheim 2019** | Apixaban 39.2%; Dabigatran 7.1%; Rivaroxaban 53.7%. | DOAC group: SAPT 58.9%, DAPT 23.0%;  VKA group: SAPT 63.2%, DAPT 20.3%. | TIA, nondisabling or disabling stroke | BARC criteria |
| **Kalogeras 2019** | Apixaban, dabigatran, edoxaban or rivaroxaban. | DOAC group: SAPT 81.4%, DAPT 17.4%;  VKA group: SAPT 62.2%, DAPT 37.7%. | N/A | BARC criteria |
| **Kawashima 2020** | Apixaban 57.7%; Dabigatran 4.0%; Edoxaban 17.2%; Rivaroxaban 21.1%. | Without additional antiplatelet treatment. | Ischemic stroke | VARC-2 criteria |
| **Mangner 2019** | Apixaban 22.5%; Dabigatran 15.9%; Edoxaban 0.5%; Rivaroxaban 61.0%. | All received clopidogrel for 6 months after TAVI. | VARC-2 criteria | VARC-2 criteria |
| **Seeger 2017** | Apixaban 2.5mg twice daily | Two groups: SAPT 66.2%, DAPT 33.8%. | Any stroke | VARC-2 criteria |

ATLANTIS: Anti-Thrombotic Strategy After Trans-Aortic Valve Implantation for Aortic Stenosis; ENVISAGE-TAVI AF: Edoxaban versus Standard of Care and Their Effects on Clinical Outcomes in Patients Having Undergone Transcatheter Aortic Valve Implantation–Atrial Fibrillation; DOAC: direct oral anticoagulant; SAPT: single antiplatelet therapy; DAPT: dual antiplatelet therapy; TAVI: Trans-Aortic Valve Implantation; TIA: Transient Ischemic Attacks; BARC: Bleeding Academic Research Consortium; ISTH: International Society for Thrombosis and Haemostasis; VARC: Valve Academic Research Consortium; N/A: not available
