# Supplementary material for: Non‐vitamin K oral anticoagulants versus vitamin K antagonists in post transcatheter aortic valve replacement patients with clinical indication for oral anticoagulation: A meta‐analysis
Source: Clin Cardiol. 2022 Feb 22;45(4):401–6. doi: 10.1002/clc.23793 (PMC9019885; doi:10.1002/clc.23793)
Supplement: Supplementary file 3 — Supporting information. [file CLC-45-401-s003.docx]

Table S2. Quality assessment based on the Newcastle–Ottawa Scale (range, 1-9)

| **Observational study** | Representativeness of exposed cohort | Selection of nonexposed cohort | Ascertainment of exposure | Absence of outcome at start of study | Comparability of cohorts | Outcome assessment | Length of follow-up | Adequacy of follow-up | NOS score |
| --- | --- | --- | --- | --- | --- | --- | --- | --- | --- |
| **Butt 2019** | 1 | 1 | 1 | 1 | 2 | 1 | 1 | 1 | 9 |
| **Jochheim 2019** | 1 | 1 | 1 | 1 | 1 | 1 | 1 | 1 | 8 |
| **Kalogeras 2019** | 1 | 1 | 1 | 1 | 1 | 1 | 1 | 1 | 8 |
| **Kawashima 2020** | 1 | 1 | 1 | 1 | 1 | 1 | 1 | 1 | 8 |
| **Mangner 2019** | 1 | 1 | 1 | 1 | 2 | 1 | 1 | 1 | 9 |
| **Seeger 2017** | 1 | 1 | 1 | 1 | 2 | 1 | 1 | 0 | 8 |

An NOS score ≥8 is considered low risk; 6-7, moderate risk; and ≤5, high risk. NOS, Newcastle–Ottawa Scale.
